# Imaging Evidence of Diabetic Choroidopathy In Vivo: Angiographic Pathoanatomy and Choroidal-Enhanced Depth Imaging

**DOI:** 10.1371/journal.pone.0083494

**Published:** 2013-12-13

**Authors:** Rui Hua, Limin Liu, Xinling Wang, Lei Chen

**Affiliations:** 1 Department of Ophthalmology, First Hospital of China Medical University, Shenyang, China; 2 Key Laboratory of Endocrine diseases in Liaoning Province, First Hospital of China Medical University, Shenyang, China; 3 Department of Ophthalmology, Fourth Hospital of China Medical University, Shenyang, China; Zhongshan Ophthalmic Center, China

## Abstract

**Purpose:**

To describe the pathoanatomy of diabetic choroidopathy (DC) in pre-diagnosed diabetic retinopathy (DR) cases and to provide angiographic and optical evidence for DC using indocyanine green angiography (ICGA) and enhanced depth imaging spectral-domain optical coherence tomography (EDI SD-OCT).

**Methods:**

A retrospective analysis of 80 eyes from 40 DR patients was conducted. In Group One, choroidal vascular abnormalities were evaluated by comparing angiographic findings from simultaneous ICGA with those from fundus fluorescein angiography (FFA). In Group Two, EDI SD-OCT was used to evaluate the subfoveal choroidal thickness (SFCT) and define the choroid boundary in order to acquire the bilateral and symmetric choroidal area (CA). Data were then analyzed by Image Pro Plus 6.0.

**Results:**

In Group One, choroidal abnormalities that were evident using ICGA but not FFA included early hypofluorescent spots in 47 eyes (75.81%), late hyperfluorescent spots in 37 eyes (59.68%), and late choroidal non-perfusion regions in 32 eyes (51.61%). In particular, a significant difference between proliferative DR (PDR) in 17 of 23 eyes (73.91%) and non-PDR in 16 of 39 eyes (41.03%) was observed in late choroidal non-perfusion regions. Eighteen of 31 eyes (58.06%) also exhibited “inverted inflow phenomena.” In Group Two, both the SFCT and CA of eyes with diabetic macular edema and serous macular detachment were significantly greater than those in the other eyes. The CA in panretinal photocoagulation (PRP) treated cases was also greater than that in non-PRP treated cases.

**Conclusions:**

Early hypofluorescent spots, late choroidal non-perfusion regions, inverted inflow phenomena, higher SFCT, and larger CA are qualitative and quantitative indexes for DC. Moreover, the late choroidal non-perfusion region is a risk factor for DC with DR. Our study suggests that the supplemental use of ICGA and EDI SD-OCT with FFA is a better choice for DR patients.

## Introduction

Diabetic choroidopathy (DC) is defined as various choroidal abnormalities in diabetics. The obliterated retinal zones in normal diabetic retinas typically present as diffuse background fluorescence. In DC, any of the following are possible: hyperfluorescent lobules in the choriocapillaris, hypofluorescent lobules from delayed filling of some focal ischemic inner choriocapillaris regions, choroidal aneurysms, neovascularizations, varicose and tortuous choroidal vessels, and late phase hypoperfusion[1,2]. Histopathologically, DC may consist of disclosed choriocapillaris and other small choroidal blood vessels with thickened basement membranes. In addition, choroidal arteries partially resemble the arteriosclerotic arteries of diabetic glomerulosclerosis, i.e., Kimmelsteil-Wilson disease[3]. The unexplained loss of visual acuity in diabetic patients who do not exhibit any retinopathy may be due to DC[4]. 

 Thus far, no systematic studies have used imaging to qualitatively and quantitatively assess DC at a pathological level in vivo. Indocyanine green angiography (ICGA) and fundus fluorescein angiography (FFA) have been used together to obtain simultaneous images of choroidal abnormalities in the same area[5]. Recently, enhanced depth imaging spectral-domain optical coherence tomography (EDI SD-OCT) has become more popular as well as acceptable for choroidal analysis, especially for cases of chronic central serous chorioretinopathy (CCSCR) [6] and for identifying subfoveal choroidal thickness (SFCT) abnormalities in patients with Vogt-Koyanagi-Harada syndrome[7].

Some pathological similarities in serous macular detachments (SMDs) exist between diabetic retinopathy (DR) and CCSCR patients. However, one study found no significant correlations between SFCT and total choroidal or subfoveal choroidal blood flow in healthy young subjects[8]. Recently, an association between a slight increase in SFCT among diabetes mellitus patients and higher glycosylated hemoglobin values has been observed[9]. No association was found between abnormal SFCTs and the presence or severity of DR, however. In this study, we introduce choroidal area (CA; µm^2^) as a new parameter for analyzing choroidal quantity by EDI SD-OCT. CA can be used to interpret the choroidal factors that contribute to SMD in non-proliferative diabetic retinopathy (NPDR) patients with diabetic macular edema (DME). In addition, CA can also be used to quantitate choroids in diabetic eyes.

## Materials and Methods

### Patients

A retrospective study was conducted in the ophthalmology outpatient clinic of China Medical University. DR patients were diagnosed and classified according to ETDRS standards[10]. The subjects were enrolled and then divided into Groups One and Two for either a qualitative and quantitative study, respectively. Patients with systematic diseases were excluded from this study. These diseases included hypertension, systemic lupus erythematosis, anemia, leukemia, and other ocular diseases, such as tractional retinal detachment (TRD) or exudate lesions. The study included 80 eyes from 40 patients. These patients included 17 males and 23 females and had a mean age of 53.78±11.50 years (range: 36-74 years). 

Group One consisted of 62 eyes from 31 DR cases, which were qualitatively assessed. This group included 13 eyes with slight NPDR, 11 with moderate NPDR, 15 with severe NPDR, and 23 with proliferative diabetic retinopathy (PDR).

Group Two consisted of 18 DME eyes from nine NPDR cases, which were quantitatively assessed for choroids. In each case, one eye had SMD and was placed in the SMD sub-group while the other eye did not have SMD and was placed in the non-SMD sub-group. Three of the nine cases in Group Two had a history of biocular panretinal photocoagulation (PRP) and were designated as the PRP-treated sub-group. The other 6 cases were placed in the non-PRP treated sub-group.

The study adhered to the tenets of the Declaration of Helsinki and was approved by the Medical Research Ethics Committee of First Hospital of China Medical University. Written informed consent was obtained from all participants.

### Methods

**Figure 1:**
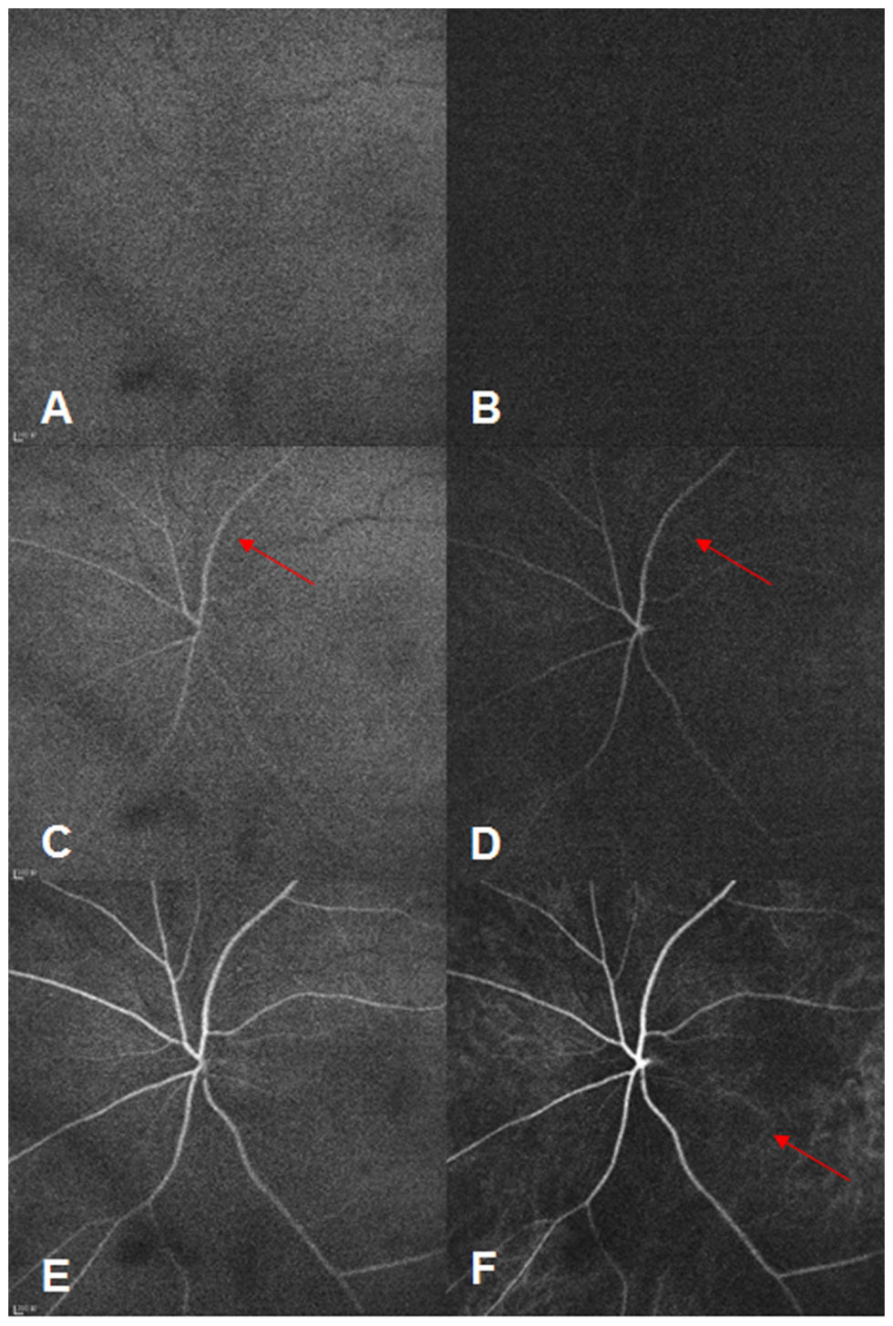
Inverted inflow phenomena. At 18.96 s, no dye was detected by FFA (A) and ICGA (B). At 20.00 s, retinal arteries (red arrow) started to fill in both FFA (C) and ICGA (D) images, without the perfusion of choroidal vessels in ICGA (D). At 21.28 s, ICGA (F) showed the filling phase of choroidal vessels (red arrow), and FFA (E) showed the filling phase of retinal vessels at the same time.

All patients received indirect ophthalmoscopy and slit-lamp fundus biomicroscopy. In Group One, ICGA (excitation 787 nm; emission 800 nm; ﬁeld of view: 30°*30°; image resolution: 768*768 pixels; dye: indocyanine green) and FFA (excitation 488 nm; emission 500 nm; ﬁeld of view: 30°*30°; image resolution: 768*768 pixels; dye: fluorescein sodium) were performed simultaneously using a Heidelberg multi-modality imaging system (Spectralis HRA+OCT; Heidelberg Engineering). Movie mode, beginning at 30 seconds, was used to record 31 eyes (six eyes with slight NPDR, six with moderate NPDR, eight with severe NPDR, and 11 with PDR) and observe whether “inverted inflow phenomena” (Figure 1), i.e., whether choroidal vessel filling time (CVFT) was longer than retinal vessel filling time (RVFT), occurred. Late stages occurred between 36 to 40 minutes. Angiographic findings from both methods were compared to identify choroidal vascular abnormalities, including: 1) early hypofluorescent spots, due to a delay in dye filling or choriocapillaris defects; 2) late hyperfluorescent spots, resulting from nodules in the choriocapillaris/stroma, choroidal aneurysms, or intrachoroidal microvascular abnormalities; and 3) late choroidal non-perfusion regions, secondary to choroidal vascular occlusion.

In Group Two, EDI SD-OCT, consisting of 1024 A scans per line (Spectralis Acquisition and Viewing Modules, version 5.3.2; Heidelberg Engineering), was used to evaluate SFCT and define the choroidal boundary between the outer retinal pigment epithelium (RPE) and inner scleral borders. 

### Choroidal area

**Figure 2:**
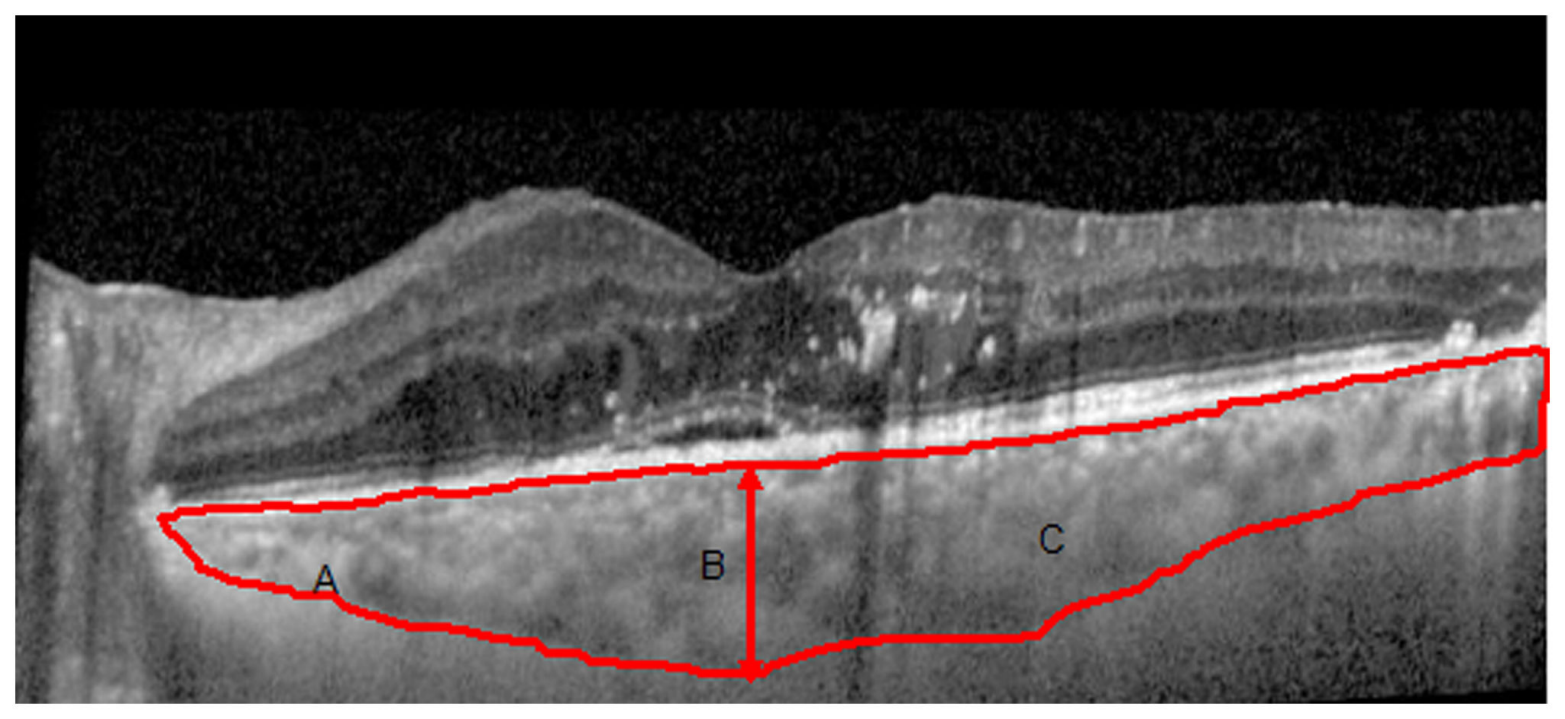
EDI SD-OCT of SMD cases. (A) inner scleral border line; (B) subfoveal choroidal thickness; (C) choroidal area (red boundaries).

A 30° horizontal EDI SD-OCT scan (6 mm) through the center of the fovea was used to acquire the choroidal boundaries. In this study, CA was defined as the region between the outer RPE and inner scleral borders (the choroidal boundary) and the vertical line drawn at the end of the choroidal boundary (three lines in all; Figure 2). CA was then measured in unit pixels (Image Pro Plus 6.0) and converted into µm^2^ using a factor of 18. The factor of 18 was the result of a comparison between Image Pro Plus 6.0 and Heidelberg Eye Explorer. Image Pro Plus has been widely used as a measuring tool for choroidal neovascularization[11], retinal vessels[12], retinal ganglion cells[13], diabetic retinopathy[14], and glaucoma[15].

### Statistical analyses

Statistical analyses were performed using SPSS, version 14.0. The data were expressed as the median (min-max). The Pearson Chi-square test was used to assess DC severity. The relationship between DR and “inverted inflow phenomena” was evaluated by Spearman rank correlations. The bilateral ocular differences between SFCT and CA were compared using Wilcoxon matched-pairs signed-ranks tests. Mann-Whitney-Wilcoxon tests were performed to analyze CA differences between the PRP-treated and non-PRP treated groups. A P value <0.05 was considered statistically significant.

## Results

### Morphological DC changes (Group One)

**Figure 3:**
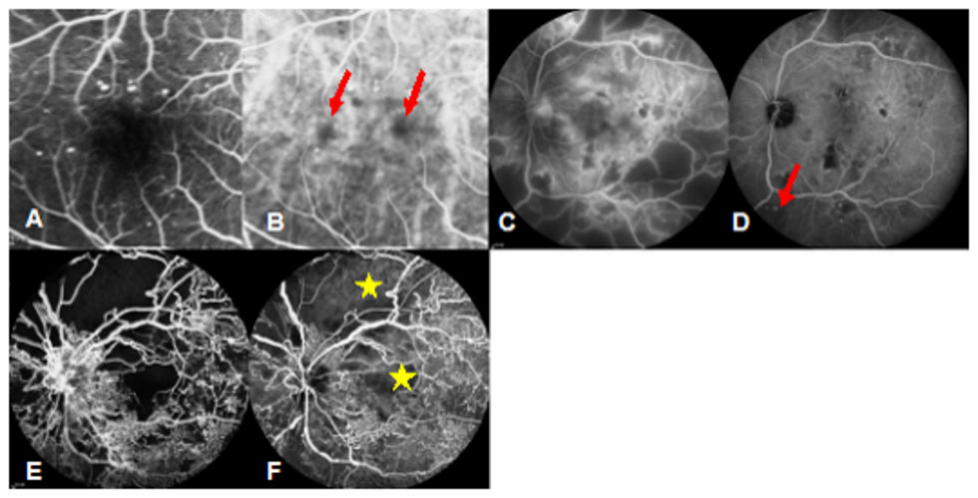
Choroidal abnormalities. Early and late hypofluorescent spots were observed by ICGA (B and D, red arrow) but not by FFA (A and C). Late choroidal non-perfusion regions were also observed by ICGA (F, yellow stars), which was consistent with the dark background fluorescence of FFA (E).

Compared to FFA, choroidal abnormalities detected by ICGA alone included early hypofluorescent spots in 47 (75.81%) eyes, late hyperfluorescent spots in 37 (59.68%) eyes, and late choroidal non-perfusion regions in 32 eyes (51.61%; Figure 3). 

The incidence rate of late choroidal non-perfusion regions detected by ICGA was 73.91% (17 of 23 eyes) for PDR, which was significantly larger than the incidence of 41.03% (16 of 39 eyes) for NPDR (Χ^2^=:6.285, P=0.012).

Additionally, 18 of 31 (58.06%) eyes exhibited inverted inflow phenomena. This included one eye (16.67%) with slight NPDR, three (50.00%) with moderate NPDR, five (62.50%) with severe NPDR, and nine (81.82%) with PDR. Inverted inflow phenomena had a positive relationship with DR severity (r=0.460, P=0.009). 

### Changes in SFCT and CA indicated DC

In Group Two, both the SFCT (364 μm [250-400 μm]) and CA (906,246 μm^2^ [571,500-996,030 μm^2^]) in SMD eyes with DME was significantly greater than in other eyes (P<0.05; Table 1). The CA in PRP-treated cases (947,448 μm^2^ [786,294-996,030 μm^2^]) was larger than that in non-PRP treated cases (777,474 μm^2^ [255,240-970,200 μm^2^]; Z=-2.435, P=0.015). In contrast, the SFCT was only marginally significantly different (Z=-1.968, P=0.049) between PRP-treated cases (365μm [276-400μm]) and non-PRP treated cases (288μm [142-397μm]).

**Table 1:** Subfoveal choroidal thickness and choroidal area (SMD and non-SMD sub-group).

Sub-group	Number of eyes	Subfoveal choroidal thickness (μm)^a^	choroidal area (μm^2^)^b^
SMD	9	364 (250-400)	906,246 (571,500-996,030)
Non-SMD	9	276 (142-351)	798,066 (255,240-906,876)

^a^ SMD compared with non-SMD: z=-2.668, P=0.008<0.05. b. SMD compared with non-SMD: z=-2.310, P=0.021<0.05.

## Discussion

Phosphatase enzyme histochemistry can quantify the loss of choriocapillaris in DC[16]. This has resulted in new insights into the causes of occlusions. Neuronal nitric oxide synthase in the parasympathetic perivascular nerve fiber of choroids can also result in diabetes-induced neuronal disorders. Thus, DC encompasses both diabetic neuropathy and microangiopathy[17].

Ischemia and delays in filling choriocapillaris can lead to early hypofluorescent spots[3,18,19,20]. In this study, the late phase choroidal non-perfusion regions detected by ICGA were significantly associated with DR severity. These regions are indicative of choroidal vascular occlusions. This is consistent with a previous study that has suggested that the association between late large hyperfluorescent spots and glycosylated hemoglobin levels could be an indicator for intrachoroidal microvascular abnormalities[21].

Another finding of this study was a positive trend between inverted inflow phenomena and DR severity. The occlusion of choroidal vessels may disturb the filling speeds of both the retinal and choroidal inflow systems. In diabetes mellitus patients, higher glucose plasma levels increased the resistance index (RI) of ocular/choroidal blood flow[22]. Similarly, measures of blood velocity in the short posterior ciliary, central retinal, and ophthalmic arteries of diabetics have suggested that the RI increased in choroidal vessels and/or the diameter of ophthalmic arteries decreased[23]. In contrast, retinal vascular resistance was normal in patients without retinopathy. To the best of our knowledge, RI represents arterial compliance[24]. Thus, it can be inferred that, in our study, choroidal vascular resistance aggravated ocular/retinal ischemia and contributed to inverted inflow phenomena. Moreover, some choroidal circulatory disorders may occur before DR. For example, choroidal blood flow deficit can be an early pathological change in DR[25].

The present study also establishes a new model for quantitatively assessing choroids by EDI SD-OCT. This model uses CA as a supply parameter for SFCT to describe choroidal vascular abnormalities. We were able to apply this model to eyes with NPDR, DME, as well as SMD.

Evidence from several studies suggests that choroidal factors may induce SMD in DR. First, the damage seen in outer blood retinal barrier has been associated with choroidal ischemia[26]. Second, some cases of DME with SMD also have macular ischemia, and choroids are primarily responsible for macular oxygen and metabolism[27]. Third, choriocapillary obstruction has been histopathologically observed in some cases of DM[3,28,29]. Finally, in CCSCR patients, the SFCT markedly increased, indicating enhanced choroidal permeability and elevated hydrostatic press, which results in SMD[6].

Larger CAs were also observed in the PRP vs. non-PRP sub-group. Mean choroidal blood flow has been shown to increase after PRP in NPDR and non-severe PDR patients[30]. This may be a possible explanation for the observations made in this study. This study also had limitations, however. For example, we did not measure the choroidal volume to analyze DC at this time. In addition, the number of patients with DC who were quantitatively assessed was slightly low. Thus, more research is needed.

In summary, early hypofluorescent spots, late phase choroidal non-perfusion regions, inverted inflow phenomena, higher SFCT, and larger CA are qualitative and quantitative indexes for DC. Moreover, late choroidal non-perfusion regions are a risk factor for DC with DR, and inverted inflow phenomenon was positively associated with DR severity. To our knowledge, this study not only certified the use of changes in choroidal thickness as a measure of DC but also suggested and applied CA for the first time. Measuring CA is an innovative strategy for qualitative and quantitative choroidal studies. Thus, our study suggests that the combined use of ICGA and EDI SD-OCT, as a supplement to FFA, is a better choice for DR patients.

## References

[1] Freyler H, Prskavec F, Stelzer N (1986) diabetic choroidopathy--a retrospective fluorescein angiography study. Preliminary report. Klin Monbl Augenheilkd 189: 144-147. doi:10.1055/s-2008-1050772. PubMed: 2429016. 2429016

[2] Saracco JB, Gastaud P, Ridings B, Ubaud CA (1982) Diabetic choroidopathy (author's transl). Fr Ophtalmol 5: 231-236. 7108132

[3] Hidayat AA, Fine BS (1985) Light and electron microscopic observations of seven cases. Diabetic choroidopathy. Ophthalmology 92: 512-522. doi:10.1016/S0161-6420(85)34013-7. PubMed: 2582331. 2582331

[4] Cao J, McLeod DS, Merges CA, Lutty GA (1998) Choriocapillaris degeneration and related pathologic changes in human diabetic eyes. Arch Ophthalmol 116: 589–597. doi:10.1001/archopht.116.5.589. PubMed: 9596494. 9596494

[5] Freeman WR, Bartsch DU, Mueller AJ, Banker AS, Weinreb RN (1998) Simultaneous indocyanine green and fluorescein angiography using a confocal scanning laser ophthalmoscope. Arch Ophthalmol 116: 455–463. doi:10.1001/archopht.116.4.455. PubMed: 9565042. 9565042

[6] Imamura Y, Fujiwara T, Margolis R, Spaide RF (2009) Enhanced depth imaging optical coherence tomography of the choroid in central serous chorioretinopathy. Retina 29: 1469-1473. doi:10.1097/IAE.0b013e3181be0a83. PubMed: 19898183. 19898183

[7] Iida T (2011) Pathophysiology of macular diseases--morphology and function. Nihon Ganka Gakkai Zasshi 115: 238-274. PubMed: 21476310. 21476310

[8] Sogawa K, Nagaoka T, Takahashi A, Tanano I, Tani T et al. (2012) Relationship Between Choroidal Thickness and Choroidal Circulation in Healthy Young Subjects. Am J Ophthalmol 153: 1129-1132. doi:10.1016/j.ajo.2011.11.005. PubMed: 22310083. 22310083

[9] Xu J, Xu L, Du KF, Shao L, Chen CX et al. (2013) Subfoveal Choroidal Thickness in Diabetes and Diabetic Retinopathy. Ophthalmology 120: 2023-2028. doi:10.1016/j.ophtha.2013.03.009. PubMed: 23697958. 23697958

[10] Early Treatment Diabetic Retinopathy Study Research Group (1991) Grading diabetic retinopathy from stereoscopic color fundus photographs--an extension of the modified Airlie House classification. ETDRS report number 10. Ophthalmology 98: 786-806. 2062513

[11] Toma HS, Barnett JM, Penn JS, Kim SJ (2010) Improved assessment of laser-induced choroidal neovascularization. Microvasc Res 80: 295-302. doi:10.1016/j.mvr.2010.05.011. PubMed: 20553963. 20553963 PMC3390000

[12] Li S, Li T, Luo Y, Yu H, Sun Y et al. (2011) Retro-orbital injection of FITC-dextran is an effective and economical method for observing mouse retinal vessels. Mol Vis 17: 3566-3573. PubMed: 22219652. 22219652 PMC3250377

[13] Salinas-Navarro M, Jiménez-López M, Valiente-Soriano FJ, Alarcón-Martínez L, Avilés-Trigueros M et al. (2009) Retinal ganglion cell population in adult albino and pigmented mice: a computerized analysis of the entire population and its spatial distribution. Vision Res 49: 637-647. doi:10.1016/j.visres.2009.01.010. PubMed: 19948111. 19948111

[14] Neubauer AS, Rothschuh A, Ulbig MW, Blum M (2008) Digital fundus image grading with the non-mydriatic Visucam(PRO NM) versus the FF450(plus) camera in diabetic retinopathy. Acta Ophthalmol 86: 177-182. doi:10.1111/j.1600-0420.2007.01029.x. PubMed: 17944975. 17944975

[15] Stroman GA, Stewart WC, Golnik KC, Curé JK, Olinger RE (1995) Magnetic resonance imaging in patients with low-tension glaucoma. Arch Ophthalmol 113: 168-172. doi:10.1001/archopht.1995.01100020050027. PubMed: 7864748. 7864748

[16] Lutty GA, McLeod DS (2005) Phosphatase enzyme histochemistry for studying vascular hierarchy, pathology, and endothelial cell dysfunction in retina and choroid. Vision Res 45: 3504-3511. doi:10.1016/j.visres.2005.08.022. PubMed: 16213000. 16213000 PMC4928484

[17] Sakurai M, Higashide T, Takeda H, Shirao Y (2002) Characterization and diabetes-induced impairment of nitric oxide synthase in rat choroid. Curr Eye Res 24: 139-146. doi:10.1076/ceyr.24.2.139.8163. PubMed: 12187486. 12187486

[18] Fukushima I, McLeod DS, Lutty GA (1997) Intrachoroidal microvascular abnormality: A previously unrecognized form of choroidal neovascularization. Am J Ophthalmol 124: 473–487. PubMed: 9323938. 9323938 10.1016/s0002-9394(14)70863-3

[19] Ito YN, Mori K, Young-Duvall J, Yoneya S (2001) Aging changes of the choroidal dye filling pattern in indocyanine green angiography of normal subjects. Retina 21: 237–242. doi:10.1097/00006982-200106000-00007. PubMed: 11421013. 11421013

[20] Shiraki K, Moriwaki M, Kohno T, Yanagihara N, Miki T (1999) Age- related scattered hypofluorescent spots on late-phase indocyanine green angiograms. Int Ophthalmol 23: 105–109. doi:10.1023/A:1026571327117. PubMed: 11196117. 11196117

[21] Shiragami C, Shiraga F, Matsuo T, Tsuchida Y, Ohtsuki H (2002) Risk factors for diabetic choroidopathy in patients with diabetic retinopathy. Graefes Arch Clin Exp Ophthalmol 240: 436-442. doi:10.1007/s00417-002-0451-5. PubMed: 12107509. 12107509

[22] Kawagishi T, Nishizawa Y, Emoto M, Konishi T, Maekawa K et al. (1995) Impaired retinal artery blood flow in IDDM patients before clinical manifestations of diabetic retinopathy. Diabetes Care 18: 1544-1549. doi:10.2337/diacare.18.12.1544. PubMed: 8722049. 8722049

[23] Tamaki Y, Nagahara M, Yamashita H, Kikuchi M (1993) Blood velocity in the ophthalmic artery determined by color Doppler imaging in normal subjects and diabetics. Jpn J Ophthalmol 37: 385-392. PubMed: 8145383. 8145383

[24] Legarth J, Nolsoe C (1990) Doppler blood velocity waveforms and the relation to peripheral resistance in the brachial artery. J Ultrasound Med 9: 449-453. PubMed: 2204716. 2204716 10.7863/jum.1990.9.8.449

[25] Muir ER, Rentería RC, Duong TQ (2012) Reduced ocular blood flow as an early indicator of diabetic retinopathy in a mouse model of diabetes. Invest Ophthalmol Vis Sci 53: 6488-6494. doi:10.1167/iovs.12-9758. PubMed: 22915034. 22915034 PMC4045095

[26] Kaur C, Foulds WS, Ling EA (2008) Blood-retinal barrier in hypoxic ischaemic conditions: basic concepts, clinical features and management. Prog Retin Eye Res 27: 622- 647. doi:10.1016/j.preteyeres.2008.09.003. PubMed: 18940262. 18940262

[27] Koleva-Georgieva D, Sivkova N (2009) Assessment of serous macular detachment in eyes with diabetic macular edema by use of spectral-domain optical coherence tomography. Graefes Arch Clin Exp Ophthalmol 247: 1461-1469. doi:10.1007/s00417-009-1124-4. PubMed: 19547995. 19547995

[28] McLeod DS, Lutty GA (1994) High-resolution histologic analysis of the human choroidal vasculature. Invest Ophthalmol Vis Sci 35: 3799-3811. PubMed: 7928177. 7928177

[29] Lutty GA, Cao J, McLeod DS (1997) Relationship of polymorphonuclear leukocytes to capillary dropout in the human diabetic choroid. Am J Pathol 151: 707-714. PubMed: 9284819. 9284819 PMC1857840

[30] Mori F, Yokota H, Nagaoka T, Konno S, Kagokawa H et al. (2003) Pulsatile ocular blood flow is unaffected in type 2 diabetes mellitus. Jpn J Ophthalmol 47: 621-622. doi:10.1016/j.jjo.2003.08.004. PubMed: 14636858. 14636858

